# Factors influencing temporal patterns in crime in a large American city: A predictive analytics perspective

**DOI:** 10.1371/journal.pone.0205151

**Published:** 2018-10-24

**Authors:** Sherry Towers, Siqiao Chen, Abish Malik, David Ebert

**Affiliations:** 1 Simon A. Levin Mathematical, Computational and Modeling Sciences Center, Arizona State University, Tempe, Arizona, United States of America; 2 VACCINE Department of Homeland Security Center of Excellence, Purdue University, West Lafayette, IN, United States of America; Victoria University of Wellington, NEW ZEALAND

## Abstract

**Background:**

Improving the accuracy and precision of predictive analytics for temporal trends in crime necessitates a good understanding of the how exogenous variables, such as weather and holidays, impact crime.

**Methods:**

We examine 5.7 million reported incidents of crime that occurred in the City of Chicago between 2001 to 2014. Using linear regression methods, we examine the temporal relationship of the crime incidents to weather, holidays, school vacations, day-of-week, and paydays. We correct the data for dominant sources of auto-correlation, and we then employ bootstrap methods for model selection. Importantly for the aspect of predictive analytics, we validate the predictive capabilities of our model on an independent data set; model validation has been almost universally overlooked in the literature on this subject.

**Results:**

We find significant dependence of crime on time of year, holidays, and weekdays. We find that dependence of aggressive crime on temperature depends on the hour of the day, and whether it takes place outside or inside. In addition, unusually hot/cold days are associated with unusual fluctuations upwards/downwards in crimes of aggression, respectively, regardless of the time of year.

**Conclusions:**

Including holidays, festivals, and school holiday periods in crime predictive analytics software can improve the accuracy and precision of temporal predictions. We also find that including forecasts for temperature may significantly improve short term crime forecasts for the temporal trends in many types of crime, particularly aggressive crime.

## Introduction

In recent years, predictive analytics and informatics software has become an essential tool for police forces nationwide to visualize and predict patterns of crime [1–7]. Necessary to the predictive performance of these tools are statistical models that take into account not only secular trends, but also various exogenous factors that might influence the incidence of different types crime; for example, climate, daylight hours, day-of-week, and holidays and festivals.

For example, many types of crimes display marked annual seasonality, as do many climate variables. Largely because of this, some theories of aggressive crime posit that hot temperatures increase irritability, stress, and aggression [8, 25]. Other theories posit that differences in routine activities and social interactions during different times of the year (or week) affect patterns in crime [26, 27]; this can be due to seasonal variations in temperature or other weather variables, or due to holidays, or the work week and weekends.

There have been several past studies of the effect of various extrinsic factors on crime, such as the effects of temperature and other climate variables [8–18], air pollution [19, 20], and holidays [21, 22], but most have examined just one, or a few, potential extrinsic factors at a time, despite the fact that exogenous factors are likely multifactorial, and also potentially inter-correlated (e.g. [8–10, 12, 14]). Many past analyses have also failed to properly take into account the significant auto-correlations in the data, or have failed to use statistical analysis and model selection methods appropriate for data with significant multicollinearities. Perhaps most important to the issue of robust predictive power of these models, model validation has been almost universally neglected in the literature, as indeed it has been in many other fields (see, for instance, References [23, 24] for a discussion of this topic). As described below, this analysis addresses each of these issues, and to the authors’ knowledege is the first analysis of this kind to address all of the issues simultaneously.

In recent years, law enforcement agencies have come to increasingly rely on quantitative analytics software packages for predicting temporal and geospatial trends in crime [28, 29]. An example of such software is the VALET package, developed by the authors and other collaborators, and used by several law enforcement agencies in the US [2, 30]. In such software packages, it is desirable to take into account what are known as “leading indicators”; factors which might forecast significant upward trends in crime in the foreseeable future [29]. For example, if factors such as climate variables, holidays, day-of-week, paydays, etc are significant leading indicators for temporal variations in crime predictive analytics methods for law enforcement may benefit from a model that incorporates these effects to more accurately and precisely predict temporal crime trends, such that policing resources can potentially be more optimally allocated [31].

While previous studies have indicated a link between, for example, violent crime and temperature, it must be cautioned that, in general, annual periodicity of a variable does not necessarily imply that the variations are directly related to annual variations in other potential explanatory variables like climate; there is a very real danger of concluding that a significant relationship exists between two independent periodic time series with the same period, simply because the two time series, even when somewhat shifted by some phase and with different sub-harmonics, will exhibit significant correlations (for a detailed discussion of this, see Reference [32]). This fact is almost universally overlooked in the literature on the topic of climate and crime, and indeed most prior analyses, including seminal analyses on the subject, did not provide plots showing the variation of crime incidence by day-of-year along with the variation of climate variables such that the temporal patterns and phases can be visually compared.

However, if climate variables *do* indeed affect crime, the following must be true in order to conclude there is an apparent significant relationship; unusual variations in climate variables like temperature (for example) should be significantly associated with unusual variations in crime. By “unusual”, we mean fluctuations beyond the typical average and variation in annual periodicity for both the crime incidence and the climate variable. Following the ansatz of [32], one can assess variations from the typical average by fitting a smooth harmonic periodic function to each of the climate variables, and considering the residuals of the fit as the fluctuations beyond the typical average annual periodicity (which we will refer to in this analysis as “periodic-corrected” data). This removes most auto-correlation from the data.

In this paper, we employ periodic-corrected data in regression analyses to examine the temporal patterns in over 5 million violent and property crimes in the City of Chicago between 2001 and 2014, and determine the relationship, if any, to

- season,
- holidays and festivals,
- day-of-week,
- paydays,
- and weather variations.

This data set is among the largest yet studied when assessing factors that may impact temporal trends in crime, and, to the authors’ knowledge, is the first analysis of its kind to examine the potential effect of climate and holidays from a predictive analytics perspective.

The climate variables that we examine are

- temperature,
- humidity
- wind speed
- air pressure,
- and precipitation.

Previous analyses of crime have also examined the potential that certain types of crime may depend quadratically on temperature [8, 10, 33, 34]. We thus also include in our regression analysis the periodic-corrected residuals for temperature (and the square of temperature), humidity, air pressure, and wind speed, and also include a factor indicating whether or not it rained. Because days with precipitation tend to be more humid and have lower air pressure than days without, we include precipitation both as an additive factor, and a factor multiplying humidity and air pressure.

To ensure robust model predictions in the presence of multicollinearities in the potential descriptive variables, we employ bootstrapping methods for model selection. We also validate the performance of the model on an independent sample of data. To the authors’ knowledge, this is the first analysis of its kind to employ either of these analysis methods.

In the following sections we describe the data sets and statistical modeling methods used in this analysis, followed by a presentation of results and discussion.

## Materials and methods

### Data

Details on all reported incidents of crime that have occurred in the City of Chicago from 2001 to present are publicly available from the Chicago Police Department’s CLEAR (Citizen Law Enforcement Analysis and Reporting) system, at data.cityofchicago.org. The data include the date, time, GPS location of the crime, description of the location of the crime, the type of crime classified according to the Uniform Crime Reporting (UCR) system of the Federal Bureau of Investigation (FBI) [35], whether an arrest was made, a description of the location of the crime (for example, on the street or in a residence), and whether domestic violence was involved. Because crimes occurring in the very early hours of the morning are typically associated with people who are still awake from the day before, we shift the day by five hours such that a day is defined as the period between 5:00 AM to 4:59 AM the next calendar day [1–3, 36]. This also facilitates analyses of crime patterns on holidays, since New Year’s Eve (for example) sees a spike in many types of crime, both before midnight, and after midnight, and also is important to analyses of weekday patterns, since after midnight on Saturday night (for example) is still considered to be an extension of that same evening.

**Fig 1:**
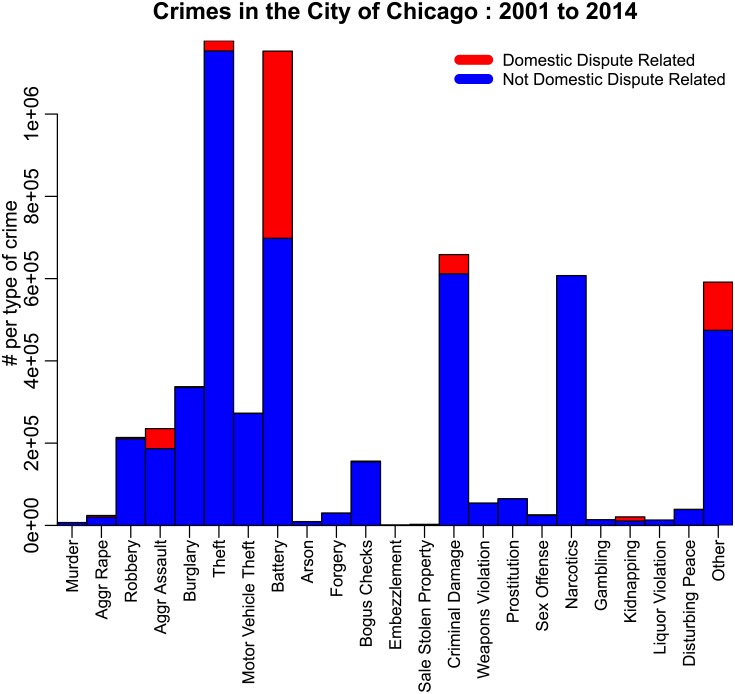
Number of crimes in the City of Chicago between 2001 to 2014, classified according to the FBI Uniform Crime Reporting system category. The number of crimes related and not related to domestic disputes are shown in red and blue, respectively. “Other” crimes include animal/elder/child abuse or neglect, harassment, parole violations, and criminal trespass.

The data from 2001 to 2014 consist of over 5.7 million reported crimes. The number of crimes by type over that time period are shown in Fig 1. In order to ensure enough data to reliably examine potential exogenous factors on crime trends with the use of linear regression models, we only examine types of crimes in Chicago that occur at least 25 times per day on average. These crimes are:

- robbery,
- aggravated assault (assault with the purpose of inflicting severe bodily injury, usually accompanied by use of a weapon, or by means likely to produce death or great bodily harm),
- burglary,
- theft,
- motor vehicle theft,
- battery (simple assault where no weapon was used, or no serious or aggravated injury resulted to the victim),
- passing of bogus checks,
- criminal damage,
- narcotics, and
- “other” crimes (includes, among other things, animal/elder/child abuse or neglect, harassment, parole violations, and criminal trespass).

These ten types of crimes account for 95% of all reported crimes.

**Fig 2:**
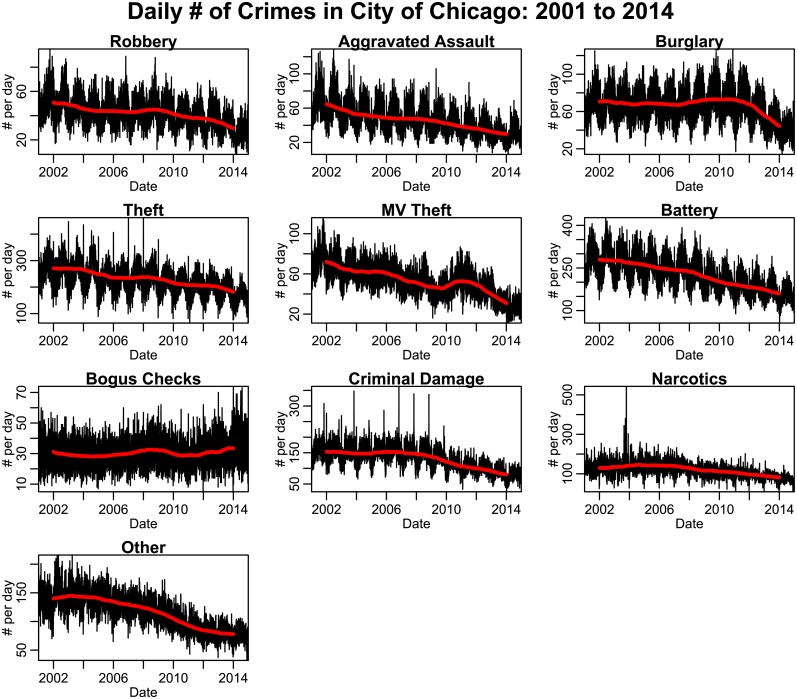
Daily number of crimes in the City of Chicago between 2001 to 2014. Overlaid in red is the long term trend estimated by the two-year running average of the data, centered around each time point. “Other” crimes include animal/elder/child abuse or neglect, harassment, parole violations, and criminal trespass.

In Fig 2, we show the daily number of these types of crimes over the 14 year period. Narcotics related crimes are among the very few types of crimes whose short term temporal trends in arrests may be significantly driven by temporal patterns in police vigilance and/or sting operations. This is likely evidenced in the spate of narcotics related arrests in 2003, as seen in Fig 2. Thus, in this analysis, we exclude narcotics crime from further consideration.

The incidence of the number of crimes by day is shown in Fig 2. As seen in Fig 2, many crime types show marked apparent seasonality. In Fig 2, we also show the running two year average of the data, in a window centered ±365 days at each time point; for most types of crimes, the long term trend is generally downward, mirroring similar trends observed across the U.S. [37, 38].

**Fig 3:**
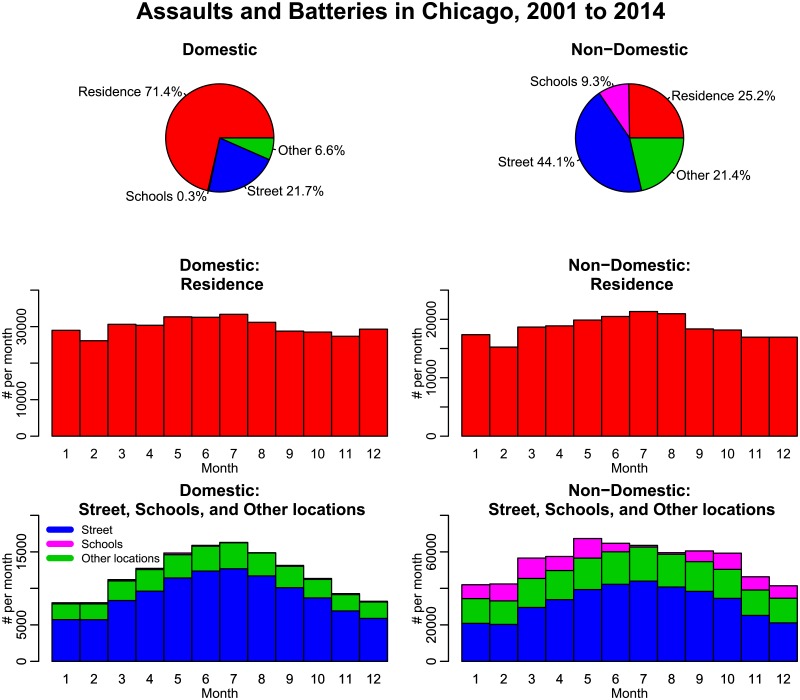
Monthly number of domestic and non-domestic aggravated assaults and batteries in the City of Chicago between 2001 to 2014, by location of occurrence. Marked seasonality is more evident in assaults and batteries occurring outside on streets compared to inside residences.

In Fig 3, we show the incidence, by month of year of domestic and non-domestic aggravated assaults and batteries in Chicago. As seen in Fig 3, the seasonality is more marked for crimes that occur outdoors on streets, sidewalks and alleys, compared to inside residences.

In this analysis we examine the effect of climate on aggravated assaults and batteries occurring on streets and in residences by time of day in five time periods, roughly divided the day into time periods meaningful for human activities like morning, afternoons, early evening, later evening, and late night.

**Morning** 5 AM to 11:59 AM,
**Afternoon** 12 PM to 4:59 PM,
**Early evening** 5 PM to 8:59 PM,
**Late evening** 9 PM to 11:59 PM, and
**Late night** 12 AM to 4:59 AM.

The afternoon time period is always daylight in Chicago no matter the time of year, and the late evening and late night time periods are always dark no matter the time of year. For the Chicago crime data, these time periods also have approximately equal incidence for assaults and batteries within the time periods.

To examine the seasonal and other periodic components, we first correct for long-term trend by subtracting the two year running average centered on each date, and adding the overall mean. Significant heteroskedasticity is observed in many of the types of crime (which, indeed, is clearly evident for some types of crime in Fig 2). We thus apply the appropriate Box-Cox transformation to each of the data sets. We refer to the end result as the “trend-corrected data”.

Due to the two-year running average associated with this trend correction, we only consider the trend-corrected data between 2002 to 2013, since a centered running average cannot be calculated for the years 2001 and 2014.

**Fig 4:**
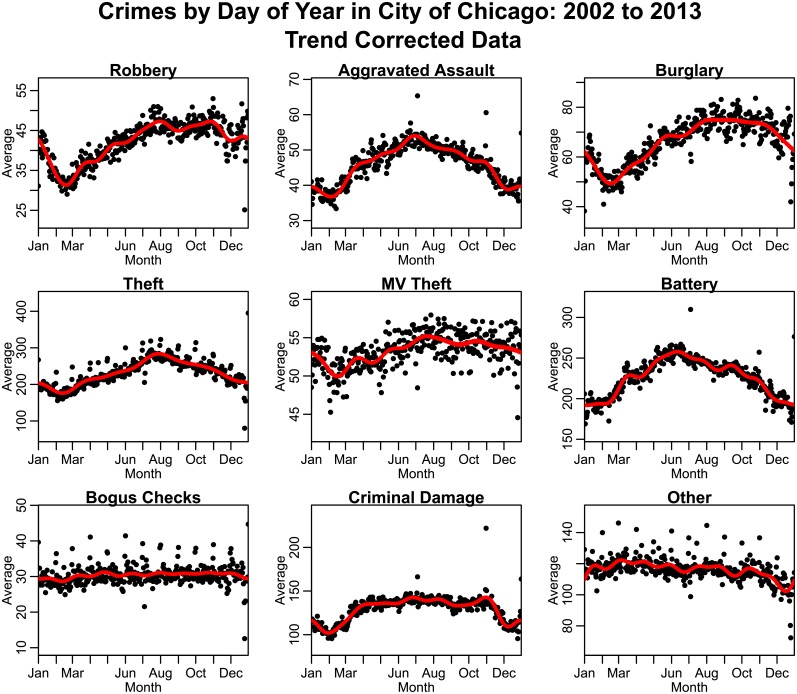
Average number of crimes by day-of-year in the City of Chicago between 2002 to 2013, after correction for long-term trends (crimes during February 29^th^ on leap years are excluded). “Other” crimes include animal/elder/child abuse or neglect, harassment, parole violations, and criminal trespass. A “day” is defined as 5 AM on a particular day of the year to 4:59 AM the next day. Thus crimes on December 31^st^, for example, include crimes committed in the early morning of January 1^st^.

In Fig 4, we show the average of the trend-corrected data by day-of-year for each type of crime. We exclude days corresponding to February 29, and in leap years we subtract one from the day-of-year for dates after February 29. We note that for most types of crime, significant day-of-year effects are evident, with many types of crime having an annual minimum incidence around February, and annual maximum incidence in the summer. As seen in Fig 4, Many types of crime also show significant fluctuations associated with regular holidays such as New Year’s Eve, Independence Day, and Christmas.

**Fig 5:**
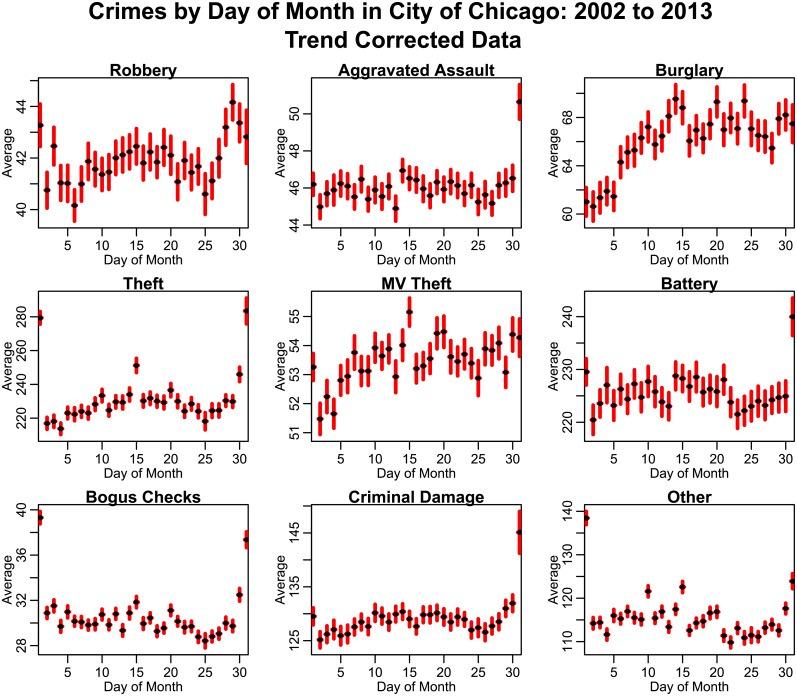
Average number of crimes by day-of-month in the City of Chicago between 2002 to 2013, after correction for long-term trends. The red error bars represent the standard error on the mean. “Other” crimes include animal/elder/child abuse or neglect, harassment, parole violations, and criminal trespass.

**Fig 6:**
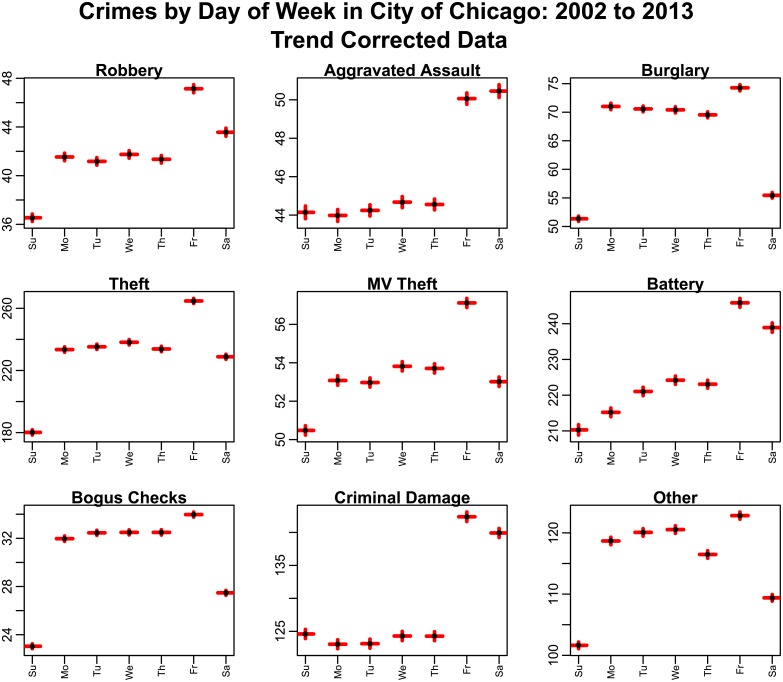
Average number of crimes by day-of-week in the City of Chicago between 2002 to 2013, after correction for long-term trends. The red error bars represent the standard error on the mean. “Other” crimes include animal/elder/child abuse or neglect, harassment, parole violations, and criminal trespass.

In Figs 5 and 6, we show the average number of crimes by day-of-month and day-of-year, respectively. Significant day-of-month effects appear to be evident for many types of crime, particularly on the first, last, and/or 15^th^ day of the month, which correspond to paydays for many workers in the population (see, for instance, the United States Bureau of Labor Statistics discussion of payday cycles at www.bls.gov/opub/btn/volume-3/how-frequently-do-private-businesses-pay-workers.htm). Significant day-of-week effects are also evident for all types of crime.

**Fig 7:**
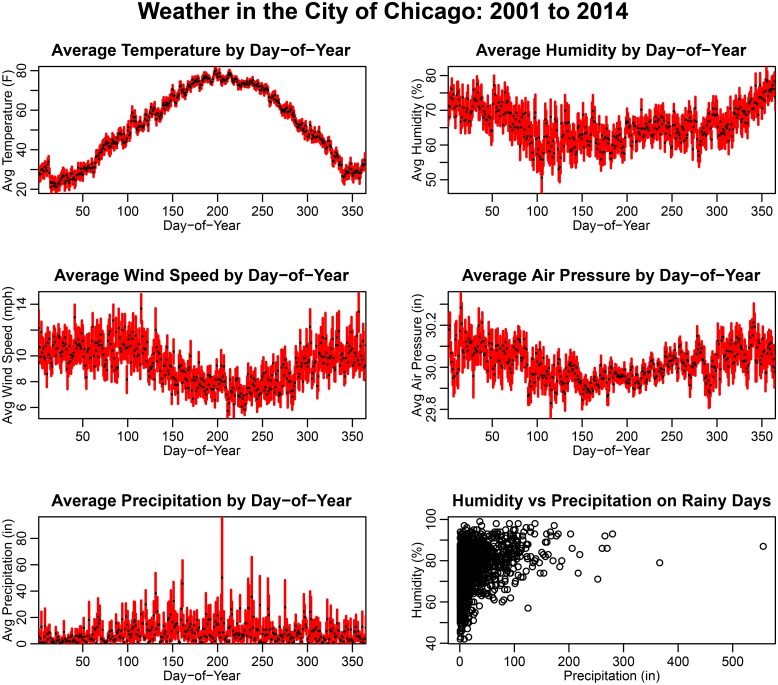
Climate data in the City of Chicago between 2001 to 2014, averaged by day-of-year. The error bars represent the standard error on the mean. The lower right hand plot shows the distribution of relative humidity vs precipitation on rainy days.

The time series of daily average temperature, humidity, wind speed, precipitation and air pressure data were obtained from the weather station at Midway International Airport, available from the Weather Underground website www.wunderground.com. Hourly weather data were obtained from the National Oceanic and Atmospheric Administration, www.noaa.gov. In Fig 7 we show the average of the weather variables by day-of-year. In Table 1 we show the average of each climate variable within each of the four seasons of the year.

**Table 1:** Average, within season, of the climate variables examined in this analysis.

	Average within season			
	Spring	Summer	Autumn	Winter
Min Temperature (°F)	42 ± 12	66 ± 6	47 ± 13	22 ± 11
Max Temperature (°F)	59 ± 15	82 ± 7	62 ± 15	34 ± 11
Avg Temperature (°F)	50 ± 13	74 ± 7	55 ± 13	28 ± 10
Avg Humidity (%)	63 ± 15	63 ± 11	65 ± 12	72 ± 11
Avg Air Pressure (in)	30.0 ± 0.2	29.9 ± 0.1	30.0 ± 0.2	30.1 ± 0.2
Avg Wind Speed (mph)	10.20 ± 3.90	7.80 ± 2.70	8.90 ± 3.70	10.40 ± 3.70
Avg Precipitation (in)	0.10 ± 0.21	0.12 ± 0.13	0.08 ± 0.20	0.05 ± 0.25

### Statistical modeling methods

In the previous section describing the sources of data, we noted that most types of crime show significant trend between 2002 to 2013, along with apparent seasonality, and dependence on holidays, day-of-week, and paydays. We also noted marked seasonality in most of the climate variables.

Thus, as described in detail in the sub-sections below, we use regression methods to analyze the daily crime data incidence data using the following steps, briefly summarized here:

- As described in the Data section, we correct for long-term trend in the daily crime incidence data by subtracting the two year centered running average, and adding the overall mean. We then apply the appropriate Box-Cox transformation to correct for heteroskedasticity. This yields the “trend-corrected” data.
- As we describe below in the following section, we correct the trend-corrected data for smoothly varying annual periodicity using harmonic linear regression methods, yielding the “periodic- and trend-corrected” data.
- As we describe below, we regress the periodic- and trend-corrected data on factors corresponding to holidays and festivals, day-of-week, and paydays. We refer to the crime data corrected for trend, annual periodicity, holidays, day-of-week, and paydays as the “holiday-, periodic-, and trend-corrected data”.
- As we describe below, we correct the climate data for smooth periodic variation, and we then additionally regress the crime holiday-, periodic-, and trend-corrected data on the climate periodic-corrected data.

To ensure robust model predictions in the presence of potential multicollinearities in the descriptive variables, we employ bootstrapping methods for model selection [39], to obtain the most parsimonious model with optimal predictive power. To determine which descriptors are needed in the fit to adequately describe the trend-corrected data without over fitting to statistical fluctuations, we perform model selection via a procedure where samples of the same size of the original data are randomly selected from the data, with replacement, 100 times, and the variables in the model which provide the lowest Aikike Information Criterion (AIC) are determined at each iteration of this bootstrapping procedure [40]. The final model including the effects of trend, smooth annual periodicity, holidays, day-of-week, paydays, and climate is derived from the combination of variables that appear in the 100 model selections as least 60% of the time [40].

We also validate the predictive performance of the models by training the model on two out of every three years of data, and testing the predictive performance using every third year of data. We note here that the issue of robust predictive power of these models is extremely important to assessing the performance of crime analytics software that employ such models. However, model validation has been almost universally neglected in the literature on the topic of exogenous factors affecting crime, as indeed it unfortunately has been in many other fields [23, 24].

#### Correcting crime incidence data for annual periodicity

As seen in Fig 4, many types of crimes display marked annual seasonality, and, as seen in Fig 7, most climate variables also have marked seasonal variation. However, annual periodicity of a variable does not necessarily imply that the variations are directly related to annual periodic variations in other potential explanatory variables related to things like climate [32]. For instance, the number of daylight hours (or conversely, hours of darkness) likely has an effect, as may regular seasonal patterns in employment, seasonal need of potential criminals for money, and/or seasonality in availability of targets for potential crime due to holidays or other climate-independent reasons [22, 41–44].

In this analysis, we wish to examine if unusually hot or cold days (for instance) lead to significant associated fluctuations in crime. To examine the potential of such short-term extrinsic effects of climate on crime, we must first correct both the trend-corrected crime data and the climate time series for annual periodicity, using the ansatz of [32]; annual periodicity in the crime data can arise from many different factors beyond just the climate variable(s) under examination, such as other potential unaccounted-for confounding variables. If the time series are not corrected for annual periodicity before this assessment, there is a danger of concluding that a significant relationship exists between two time series with the same period simply because the two time series, even when shifted by some phase, will exhibit significant correlations [32].

To correct a time series for annual periodicity patterns, we exploit the fact that any periodic time series can be expressed as a finite or infinite sum of sines and cosines, and use a technique known as harmonic, or trigonometric, linear regression where we model the observations, *Y*_*i*_, at times *t*_*i*_ with the predictor [45]

$$\begin{array}{c} y_{i}=A+\sum_{j=1}^{k}(B_{j}\sin\frac{j2\pi t_{i}}{T}+C_{j}\cos\frac{j2\pi t_{i}}{T}), \end{array}$$ (1)

where *T* = 365 days, *A* is the average crime incidence, *B*_*j*_ and *C*_*j*_ are regression coefficients, and *k* is sufficiently large enough to capture the overall patterns in the annual periodicity, without being so large as to be sensitive to local effects, such as holiday periods like Thanksgiving, and Christmas to New Year’s. In this analysis, we use *k* = 6, which yields sensitivity to components of the periodic variation down to two months (we note here that inclusion of harmonics with order five or seven does not change the conclusions of the analysis).

In order to examine the relative contribution of trend and annual periodicity, holidays to the variation in crime, we perform incremental model selection fits to the trend-corrected data with the model of Eq 1, performing the bootstrap model selection procedure at each iteration to determine the best-fit model. We take the residuals of the fit, plus the mean of the fit, and thus obtain the data corrected for trend and smooth annual periodicity. We refer to this as the “periodic- and trend-corrected data”.

#### Modeling the effect of holidays, day-of-week, and paydays on crime

Additional significant annual peaks or dips in crime can also be associated with holidays, festivals, or school vacation periods. Here we use the word “holiday” to refer to these events. In the regression fit to the crime data, we thus additionally include a factor with levels corresponding to the days:

- New Year’s Day,
- New Year’s Day (observed),
- Martin Luther King Jr.’s Birthday,
- Lincoln’s Birthday,
- Valentine’s Day,
- Presidents’ Day,
- Casimir Pulaski Day (a public bank holiday in Illinois celebrated the first Monday of March),
- The Saturday before St. Patrick’s day (a celebration in Chicago),
- St. Patrick’s day,
- Easter,
- Memorial Day,
- Independence Day,
- Independence Day (observed),
- Labor Day,
- Columbus Day,
- Halloween (even though this is not a public bank holiday, we note it apparently is associated with significant variations in some types of crime),
- Election Day (even years),
- Veterans’ Day,
- Thanksgiving,
- Thanksgiving Friday,
- Christmas Eve,
- Christmas, and
- New Year’s Eve.

We also include a factor corresponding to the school holiday vacation periods for days independent of other holidays and festivals. Furthermore, we include a factor with day-of-week levels, and a factor with levels corresponding to paydays on first of the month, 15^th^ of the month, and the last day of the month.

In order to examine the relative contribution of trend, annual periodicity, holidays, day-of-week, and paydays to the variation in crime, we perform incremental model selection fits to the periodic- and trend-corrected data, adding each effect one at a time, performing the bootstrap model selection procedure at each iteration to determine the best-fit model. We take the residuals of the fit, plus the mean of the fit, and thus obtain the data corrected for trend, annual periodicity, holidays, day-of-week, and paydays. We refer to this as the “holiday-, periodic-, and trend-corrected data”.

#### Modeling the effect of weather on crime

We correct the weather data for annual periodicity using the harmonic regression model of Eq 1, performing the bootstrap model selection procedure as described above. We refer to the climate data corrected for annual periodicity as the “periodic-corrected climate data”.

Previous analyses of crime have examined the potential that certain types of crime may depend both linearly and quadratically on temperature [8, 10, 33, 34]. It is a simple mathematical exercise to show that if the incidence of crime really does significantly linearly depend on a climate variable like temperature, a significant linear relationship will also be apparent when we regress the holiday-, periodic-, and trend-corrected crime data on the periodic-corrected climate data (see the discussion on this topic in [32]). Further, because the square of a periodic time series is just another periodic time series, if crime quadratically depends on temperature, we would thus observe a significant relationship between the holiday-, periodic-, and trend-corrected crime data and both the periodic-corrected temperature data, and the periodic-corrected square of the temperature data.

In order to determine if short term variations in climate variables have a significant effect on crime, we thus regress the crime holiday-, periodic-, and trend-corrected data on the climate periodic-corrected data. Because a particularly warm day in winter (for instance) may not have the same effect on crime as a particularly warm day in summer, we include as a multiplicative factor in the fit the season of the year, where December through February are considered winter, March through May are considered spring, June through August are considered summer, and September through November are considered autumn. We include in the regression the periodic-corrected residuals for temperature (and the square of temperature), humidity, air pressure, and wind speed, and also include a factor indicating whether or not it rained. Because days with precipitation tend to be more humid, and have lower air pressure, than days without, we include precipitation both as an additive factor, and a factor multiplying humidity and air pressure.

We employ the bootstrap method, described above, to obtain the most parsimonious model with optimal predictive power.

## Results

### Best-fit models

In Table 2 we show, for each type of crime considered, the *R*^2^ of the best-fit regression models that incrementally include trend, annual periodicity, holidays, day-of-week, paydays, and climate. We show this both for the training data, and also the fraction of the variance of the independent test data explained by the model predictions for that data set.

**Table 2:** The *R*^2^ of the best-fit regression models that incrementally include trend, annual periodicity, holidays, day-of-week, paydays, and climate effects. “Other” crimes include animal/elder/child abuse or neglect, harassment, parole violations, and criminal trespass. The test sample consists of every third year of data, and the training sample consists of the remaining data.

	*R*^2^ Described by Model: Training Sample								
	Robbery	Aggr Assault	Burglary	Theft	MV Theft	Battery	Bogus Checks	Criminal Damage	“Other”
Trend	0.12	0.24	0.02	0.22	0.39	0.42	0.03	0.41	0.67
Trend + Annual	0.38	0.55	0.29	0.50	0.42	0.63	0.05	0.59	0.69
Trend + Annual + Holiday	0.42	0.57	0.31	0.58	0.44	0.67	0.13	0.63	0.71
Trend + Annual + Holiday + Weekday	0.52	0.65	0.61	0.76	0.48	0.75	0.43	0.73	0.77
Trend + Annual + Holiday + Weekday + Payday	0.52	0.65	0.61	0.80	0.48	0.75	0.51	0.73	0.80
Trend + Annual + Holiday + Weekday + Payday + Climate	0.54	0.75	0.63	0.82	0.49	0.84	0.51	0.80	0.81
	*R*^2^ Described by Model: Test Sample								
	Robbery	Aggr Assault	Burglary	Theft	MV Theft	Battery	Bogus Checks	Criminal Damage	“Other”
Trend	0.14	0.31	0.22	0.23	0.57	0.46	0.01	0.50	0.70
Trend + Annual	0.38	0.58	0.44	0.54	0.58	0.69	−0.01	0.70	0.73
Trend + Annual + Holiday	0.39	0.59	0.45	0.60	0.59	0.72	0.05	0.72	0.74
Trend + Annual + Holiday + Weekday	0.49	0.66	0.69	0.76	0.62	0.77	0.33	0.78	0.79
Trend + Annual + Holiday + Weekday + Payday	0.49	0.66	0.68	0.79	0.62	0.77	0.40	0.78	0.81
Trend + Annual + Holiday + Weekday + Payday + Climate	0.51	0.74	0.70	0.80	0.61	0.84	0.41	0.84	0.81

As seen in Table 2, for all types of crime, a significant dependence on trend, annual periodic variation, holidays, and weekdays is observed in both the training and test data. The inclusion of paydays in the fit only significantly improves the description of the variation in theft, bogus checks, and “other” types of crime.

Additionally, as seen in Table 2, the only crimes for which an apparent significant improvement in *R*^2^ is observed in both the training and test data when climate variables are included in the fit are aggravated assaults, batteries, and criminal damage. The improvement in the *R*^2^ achieved by adding climate variables in the fit is only modest for robbery and burglary.

**Fig 8:**
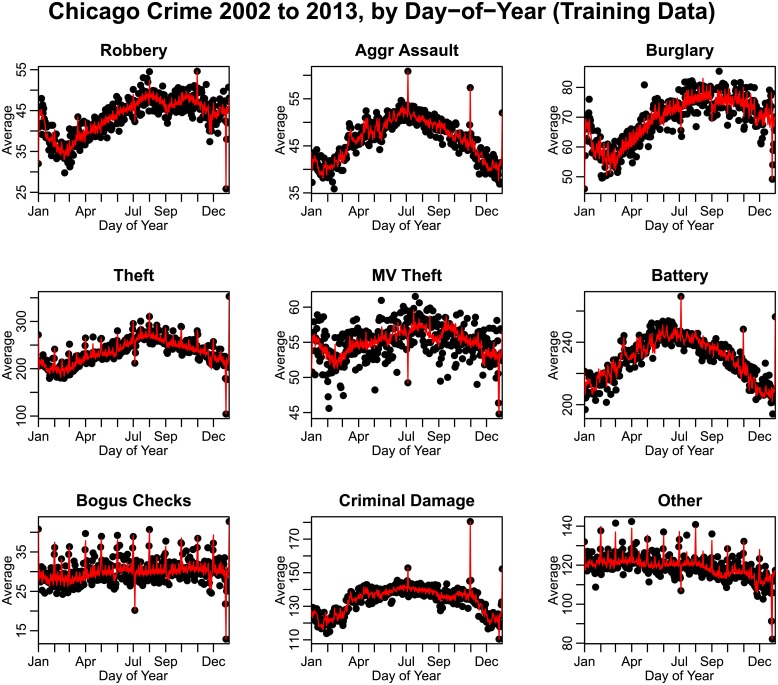
Average number of crimes by day-of-year in the City of Chicago between 2002 to 2013, with every two out of three years selected as training data for the linear regression fits. Overlaid in red are the best-fit regression models, including periodic, holiday, day-of-week, payday and climate effects. “Other” crimes include animal/elder/child abuse or neglect, harassment, parole violations, and criminal trespass.

In Fig 8 we show the results of the best-fit regression models to the training data (every two out of three years of data) that include trend, annual periodicity, holidays, day-of-week, paydays, and climate effects (where significant).

**Fig 9:**
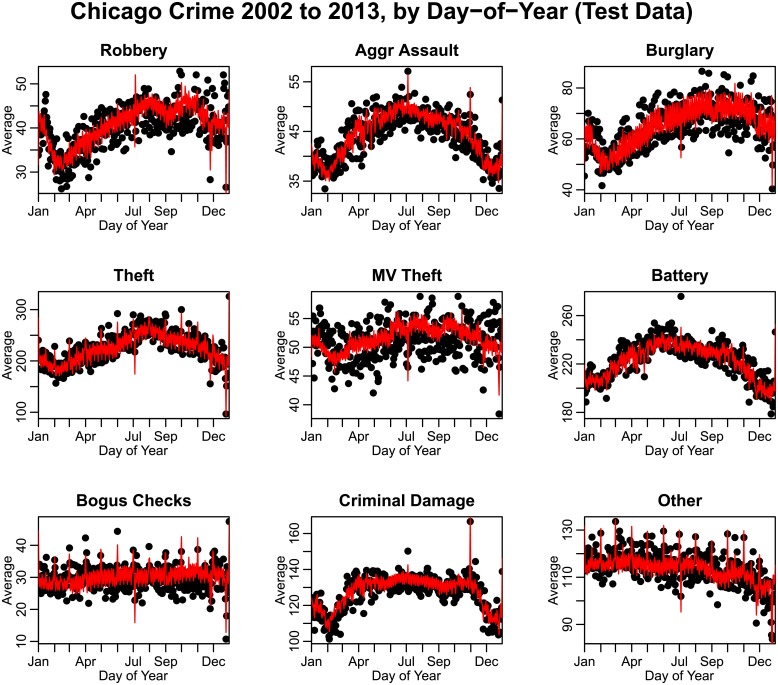
Average number of crimes by day-of-year in the City of Chicago between 2002 to 2013, with every one out of three years selected as test data for the performance of the linear regression fits to the training data. Overlaid in red are the predictions of the regression models, including periodic, holiday, day-of-week, payday and climate effects, as selected by the model selection procedure. “Other” crimes include animal/elder/child abuse or neglect, harassment, parole violations, and criminal trespass.

In Fig 9 we show the performance of the linear regression model in predicting crime in the independent test data set (every third year of data), overlaying the model predictions on the test data.

### Dependence of crime on day-of-week

In order to examine how crime depends on weekday, we take the most parsimonious model determined for each type of crime that includes trend, smooth annual periodicity, holidays, weekdays, paydays, and climate. Weekdays are included as a factor; we thus examine the variation by weekday factor level, and examine this variation relative to the fit average incidence on Wednesdays, which is a weekday when most types of crime have minimum incidence. In Table 3, we show the average resulting percentage increase or decrease in each type of crime by weekday, relative to Wednesdays, taking into account trend, annual periodicity, holidays, paydays, and climate. Because multiple tests of significance are involved, we must correct the *α* used to assess significance using the Bonferroni correction. A total of six weekdays are assessed, and nine types of crime, leading to 54 tests of significance. We thus set *α* to *α* = 0.05/54. As seen in Table 3, we find all types of crime are significantly more prevalent on Fridays. Robberies, aggravated assaults, batteries, and criminal damage are significantly more prevalent on Saturdays, and most types of crime are significantly less prevalent on Sundays.

**Table 3:** The percentage change in the rate of crime by weekday, relative to the average on Wednesdays. The numbers in bold represent values that are significant to *p* < 0.05/(6 * 9) (two-tailed *Z* test, with Bonferroni corrected *α*). “Other” crimes include animal/elder/child abuse or neglect, harassment, parole violations, and criminal trespass.

	Percentage change in average incidence of crime								
	Robbery	Aggravated Assault	Burglary	Theft	MV Theft	Battery	Bogus Checks	Criminal Damage	“Other”
Thursday	−0.1 ± 0.8	+0.2 ± 0.5	−0.3 ± 0.6	−0.7 ± 0.4	+0.1 ± 0.6	+0.2 ± 0.3	+1.0 ± 0.8	+0.2 ± 0.3	**−1.8 ± 0.4**
Friday	**+11.3 ± 0.8**	**+10.0 ± 0.5**	**+5.3 ± 0.6**	**+8.7 ± 0.4**	**+5.6 ± 0.6**	**+6.3 ± 0.3**	**+4.6 ± 0.8**	**+9.2 ± 0.3**	**+1.4 ± 0.4**
Saturday	**+3.6 ± 0.8**	**+9.9 ± 0.5**	**−18.0 ± 0.6**	**−3.3 ± 0.4**	−2.0 ± 0.6	**+3.6 ± 0.3**	**−15.6 ± 0.8**	**+8.1 ± 0.3**	**−6.2 ± 0.4**
Sunday	**−11.2 ± 0.8**	−0.9 ± 0.5	**−23.0 ± 0.6**	**−19.0 ± 0.4**	**−6.7 ± 0.6**	**−3.8 ± 0.3**	**−28.9 ± 0.8**	+0.3 ± 0.3	**−10.3 ± 0.4**
Monday	+0.2 ± 0.8	−0.9 ± 0.5	**+2.8 ± 0.7**	−0.7 ± 0.5	−1.0 ± 0.7	**−1.7 ± 0.3**	+0.1 ± 0.9	−0.6 ± 0.3	−0.8 ± 0.4
Tuesday	−1.0 ± 0.8	−0.7 ± 0.5	+0.4 ± 0.6	−0.9 ± 0.4	−1.6 ± 0.6	**−0.9 ± 0.3**	+0.1 ± 0.8	−0.5 ± 0.3	−0.5 ± 0.4

### Dependence of crime on holidays

In order to examine how crime depends on holiday, we take the most parsimonious model determined for each type of crime that includes trend, smooth annual periodicity, holidays, weekdays, paydays, and climate. Holidays are included as a factor; we thus examine the variation by holiday factor level, and examine this variation relative to the fit average incidence on non-holidays. In Table 4, we show the average percentage increase or decrease in each type of crime by holiday, taking into account trend, annual periodicity, day-of-week, paydays, and climate. There are 25 holidays examined, and nine different crimes, therefore we assess *α* as *α* = 0.05/225. For clarity of presentation, we only show results significant to *p* < *α* in Table 4.

**Table 4:** The percentage change in the average incidence of crime on holidays. For clarity, only values that are significant to *p* < 0.05/(25 * 9) are shown (two-tailed *Z* test, with Bonferroni corrected *α*). “Other” crimes include animal/elder/child abuse or neglect, harassment, parole violations, and criminal trespass.

	Percent change in average incidence of crime								
	Robbery	Aggravated Assault	Burglary	Theft	MV Theft	Battery	Bogus Checks	Criminal Damage	“Other”
New Year’s	−29.4 ± 3.1		−28.7 ± 3.5						
New Year’s (obs)									
MLK Jr.			−13.9 ± 1.8			−5.9 ± 0.6			
Lincoln’s Birthday						−8.0 ± 0.7			
Valentines									
Presidents						−11.3 ± 1.0			
Casimir Pulaski			−14.9 ± 1.3			−9.1 ± 0.7			
St Patricks (Sat)									
St Patricks									
Easter	−18.7 ± 2.2			−13.9 ± 1.6					
Memorial	−14.8 ± 1.3		−24.3 ± 1.8	−17.6 ± 2.0			−33.8 ± 2.1		
Independence		+16.4 ± 1.5	−16.1 ± 2.0	−18.0 ± 2.3		+8.1 ± 0.6	−30.9 ± 5.6	+11.6 ± 1.3	−12.8 ± 1.9
Independence (obs)			−22.2 ± 0.9						
Labor	−14.2 ± 1.5		−26.3 ± 2.2	−15.4 ± 1.7			−28.0 ± 2.7		
Columbus			−11.9 ± 1.2			−6.4 ± 0.6			
Halloween	+15.8 ± 2.1	+24.2 ± 3.2				+8.9 ± 1.0		+30.1 ± 4.0	
Election									
Veterans						−7.0 ± 0.8			
Veterans (obs)									
Thanksgiving	−38.4 ± 4.1		−15.5 ± 1.9	−37.7 ± 3.9	−15.6 ± 2.3		−54.2 ± 2.0		−18.7 ± 3.0
Thanksgiving Friday		−10.0 ± 1.1	−26.0 ± 3.1	−9.6 ± 1.0		−10.7 ± 0.9			
Christmas Eve				−16.4 ± 2.3			−25.7 ± 3.3		−20.2 ± 3.3
Christmas	−40.2 ± 5.0		−30.7 ± 4.8	−51.3 ± 8.1	−18.1 ± 2.8		−59.6 ± 10.2	−8.2 ± 1.2	−26.2 ± 4.4
New Year’s Eve		+27.5 ± 5.0		+47.4 ± 7.1		+20.6 ± 2.6	+22.7 ± 2.3	+21.7 ± 4.8	
School Vacation			−4.1 ± 0.6		+2.9 ± 0.4	−4.6 ± 0.5			−2.5 ± 0.4

As seen in Table 4, burglary goes down significantly on many holidays, as does theft (except on New Year’s Eve, when it goes up significantly), and robbery (except on Halloween, when it goes up significantly). Assaults and batteries also go significantly down on many holidays, except for Independence Day, Halloween, and New Year’s Eve.

Christmas and Thanksgiving are associated with a significant decrease in many types of crime, while New Year’s Eve is associated with a significant increase in several types of crime, including aggravated assaults and batteries.

Excluding New Year’s Eve, Halloween, and Independence Day, significant reductions in crime are associated with several types of crime and holidays, and no significant increases (except for a modest increase in motor vehicle thefts during school vacations).

### Effect of weather on crime

#### Effect of climate on crime

In Table 5 we show the percentage change in the average incidence of crime due to fluctuations in climate variables, by season of year. Battery is significantly positively associated with temperature at all times of the year. The only significantly negative association between temperature and crime is for burglary during the summer.

**Table 5:** The percentage change in the average incidence of crime due to fluctuations in climate variables. Only changes significant to *p* < 0.05/(32*6) are shown, and only crimes for which climate appears to play a significant role as assessed by the changes in *R*^2^ for the training and test samples (see Table 2) are shown.

	Percent change in average incidence of crime					
	Robbery	Aggravated Assault	Burglary	Theft	Battery	Criminal Damage
Spring, Avg Temperature (%/°F)				+0.64 ± 0.13	+0.37 ± 0.01	+0.98 ± 0.09
Summer, Avg Temperature (%/°F)			−2.69 ± 0.56		+0.33 ± 0.02	
Fall, Avg Temperature (%/°F)	+0.36 ± 0.06				+0.35 ± 0.02	+0.78 ± 0.11
Winter, Avg Temperature (%/°F)	+0.48 ± 0.04	+0.37 ± 0.09	+0.80 ± 0.14	+0.69 ± 0.09	+0.26 ± 0.01	+0.58 ± 0.06
Spring, Avg Temperature^2^ (%/°F^2^)				−0.005 ± 0.001		−0.007 ± 0.001
Summer, Avg Temperature^2^ (%/°F^2^)			+0.019 ± 0.004			
Fall, Avg Temperature^2^ (%/°F^2^)						−0.005 ± 0.001
Winter, Avg Temperature^2^ (%/°F^2^)						
Spring, Avg Humidity, not raining (%/%)					−0.06 ± 0.01	
Summer, Avg Humidity, not raining (%/%)						
Fall, Avg Humidity, not raining (%/%)						
Winter, Avg Humidity, not raining (%/%)						−0.14 ± 0.02
Spring, Avg Humidity, raining (%/%)		−0.20 ± 0.04			−0.08 ± 0.02	
Summer, Avg Humidity, raining (%/%)					−0.12 ± 0.03	
Fall, Avg Humidity, raining (%/%)					−0.14 ± 0.03	
Winter, Avg Humidity, raining (%/%)						
Spring, Avg Pressure, not raining (%/in)						
Summer, Avg Pressure, not raining (%/in)						
Fall, Avg Pressure, not raining (%/in)						
Winter, Avg Pressure, not raining (%/in)						
Spring, Avg Pressure, raining (%/in)						
Summer, Avg Pressure, raining (%/in)						
Fall, Avg Pressure, raining (%/in)						
Winter, Avg Pressure, raining (%/in)						
Spring, Avg Wind (%/mph)		−0.30 ± 0.06			−0.23 ± 0.04	
Summer, Avg Wind (%/mph)					−0.21 ± 0.05	
Fall, Avg Wind (%/mph)		−0.39 ± 0.07			−0.18 ± 0.04	
Winter, Avg Wind (%/mph)		−0.37 ± 0.07			−0.24 ± 0.04	
Spring, Raining (%)		−2.74 ± 0.68			−2.10 ± 0.38	−1.79 ± 0.47
Summer, Raining (%)		−2.86 ± 0.69				
Fall, Raining (%)						
Winter, Raining (%)				−3.46 ± 0.55	−1.55 ± 0.40	−3.42 ± 0.50

The quadratic term for temperature is significantly negatively associated with criminal damage and theft at some times of the year, and positively associated with burglary during the summer.

Wind speed is significantly negatively associated with both aggravated assaults and batteries, as is precipitation.

#### Effect of climate on assaults and batteries occurring in the street, by time of day

In Table 6 we show the percentage change in the average incidence of aggravated assaults and batteries that occur on the street, by time of day, due to fluctuations in climate variables, by season of year.

**Table 6:** The percentage change in the average incidence of aggravated assaults and batteries occurring on the street due to fluctuations in climate variables. Only changes significant to *p* < 0.05/(32*5) are shown.

	Percent change in average incidence of street aggr assaults and batteries				
	5am to 11:59am *N* = 55935	12pm to 4:59pm *N* = 114943	5pm to 8:59pm *N* = 107075	9pm to 11:59pm *N* = 75610	12am to 4:59am *N* = 73545
Spring, Avg Temperature (%/°F)	+0.88 ± 0.07	+1.95 ± 0.26	+2.11 ± 0.24		
Summer, Avg Temperature (%/°F)	+0.64 ± 0.11	+3.50 ± 0.72	+3.19 ± 0.69	+3.75 ± 0.75	
Fall, Avg Temperature (%/°F)	+0.81 ± 0.08		+1.65 ± 0.30		
Winter, Avg Temperature (%/°F)	+0.78 ± 0.07	+0.79 ± 0.17	+0.86 ± 0.17		
Spring, Avg Temperature^2^ (%/°F^2^)		−0.012 ± 0.002	−0.009 ± 0.002		+0.011 ± 0.003
Summer, Avg Temperature^2^ (%/°F^2^)		−0.022 ± 0.005	−0.02 ± 0.005	−0.02 ± 0.005	
Fall, Avg Temperature^2^ (%/°F^2^)					
Winter, Avg Temperature^2^ (%/°F^2^)					
Spring, Avg Humidity, not raining (%/%)			−0.38 ± 0.05		
Summer, Avg Humidity, not raining (%/%)					
Fall, Avg Humidity, not raining (%/%)					
Winter, Avg Humidity, not raining (%/%)			−0.28 ± 0.06		
Spring, Avg Humidity, raining (%/%)			−0.61 ± 0.06		
Summer, Avg Humidity, raining (%/%)			−0.53 ± 0.07		
Fall, Avg Humidity, raining (%/%)			−0.66 ± 0.07		
Winter, Avg Humidity, raining (%/%)			−0.59 ± 0.10		
Spring, Avg Pressure, not raining (%/in)					
Summer, Avg Pressure, not raining (%/in)					
Fall, Avg Pressure, not raining (%/in)					
Winter, Avg Pressure, not raining (%/in)					
Spring, Avg Pressure, raining (%/in)					
Summer, Avg Pressure, raining (%/in)					
Fall, Avg Pressure, raining (%/in)					
Winter, Avg Pressure, raining (%/in)					
Spring, Avg Wind (%/mph)	−0.61 ± 0.17	−0.57 ± 0.13	−0.92 ± 0.13	−0.63 ± 0.14	
Summer, Avg Wind (%/mph)					
Fall, Avg Wind (%/mph)		−0.60 ± 0.14	−0.92 ± 0.14	−0.54 ± 0.14	
Winter, Avg Wind (%/mph)		−0.97 ± 0.14	−0.79 ± 0.14	−0.53 ± 0.14	
Spring, Raining (%)		−8.98 ± 1.03		−12.63 ± 1.07	−9.69 ± 1.15
Summer, Raining (%)	−4.65 ± 1.32			−8.26 ± 1.08	−8.21 ± 1.18
Fall, Raining (%)		−5.39 ± 1.06		−9.25 ± 1.11	−7.38 ± 1.20
Winter, Raining (%)		−4.25 ± 1.08		−8.69 ± 1.13	−6.68 ± 1.23

For times between 5 AM to 8:59 PM, the incidence of assaults and batteries on the street is significantly positively associated with temperature for all seasons of the year, except during the autumn between noon to 4:59 PM. For times between 9 PM to 4:59 AM, there is no significant association, except during the summer between 9 AM to 11:59 PM.

Rain, wind, humidity, and the square of the temperature are significantly negatively associated with aggravated assaults and batteries occurring on the street.

#### Effect of climate on assaults and batteries occurring in residences, by time of day

In Table 7 we show the percentage change in the average incidence of aggravated assaults and batteries that occur in residences, by time of day, due to fluctuations in climate variables, by season of year. Unlike street crimes, temperature and assaults and batteries in residences are significantly positively associated at all times of the day, including the evening and late night hours.

**Table 7:** The percentage change in the average incidence of aggravated assaults and batteries occurring in residences due to fluctuations in climate variables. Only changes significant to *p* < 0.05/(32*5) are shown.

	Percent change in average incidence of residential aggr assaults and batteries				
	5am to 11:59am *N* = 100897	12pm to 4:59pm *N* = 107954	5pm to 8:59pm *N* = 103604	9pm to 11:59pm *N* = 89855	12am to 4:59am *N* = 91848
Spring, Avg Temperature (%/°F)	+0.14 ± 0.04			+0.47 ± 0.05	+0.34 ± 0.04
Summer, Avg Temperature (%/°F)	+0.31 ± 0.06	+0.28 ± 0.06	+0.28 ± 0.07	+0.56 ± 0.08	+0.58 ± 0.06
Fall, Avg Temperature (%/°F)	+0.27 ± 0.04	+0.27 ± 0.05	+0.36 ± 0.05	+0.43 ± 0.06	+0.34 ± 0.05
Winter, Avg Temperature (%/°F)			+0.22 ± 0.04		+0.17 ± 0.04
Spring, Avg Temperature^2^ (%/°F^2^)					
Summer, Avg Temperature^2^ (%/°F^2^)					
Fall, Avg Temperature^2^ (%/°F^2^)					
Winter, Avg Temperature^2^ (%/°F^2^)					
Spring, Avg Humidity, not raining (%/%)					
Summer, Avg Humidity, not raining (%/%)					
Fall, Avg Humidity, not raining (%/%)					
Winter, Avg Humidity, not raining (%/%)					
Spring, Avg Humidity, raining (%/%)					−0.14 ± 0.04
Summer, Avg Humidity, raining (%/%)					
Fall, Avg Humidity, raining (%/%)					
Winter, Avg Humidity, raining (%/%)					
Spring, Avg Pressure, not raining (%/in)					
Summer, Avg Pressure, not raining (%/in)					
Fall, Avg Pressure, not raining (%/in)					
Winter, Avg Pressure, not raining (%/in)					
Spring, Avg Pressure, raining (%/in)					
Summer, Avg Pressure, raining (%/in)					
Fall, Avg Pressure, raining (%/in)					
Winter, Avg Pressure, raining (%/in)					
Spring, Avg Wind (%/mph)					
Summer, Avg Wind (%/mph)					
Fall, Avg Wind (%/mph)					
Winter, Avg Wind (%/mph)					
Spring, Raining (%)					
Summer, Raining (%)					
Fall, Raining (%)					
Winter, Raining (%)					

Unlike street crimes, rain, wind, humidity, and the square of the temperature are not significantly associated with aggravated assaults and batteries occurring in residences, except for a negative association to humidity during rainy days between midnight to 4:59AM in the spring.

## Discussion

As seen in Table 2 and Figs 8 and 9, we find that a regression model that includes trend, smooth annual periodicity, holidays and weekdays has significant predictive power for all types of crime examined in this analysis (*R*^2^ ≥ 0.43 in all cases). This is in agreement with previous studies [14, 15, 21].

We find that inclusion of climate variables (particularly temperature) only significantly improves the predictive power of the statistical regression models for aggravated assaults, batteries, and criminal damage; motor vehicle theft, bogus checks, and “other” crime (including animal/elder/child abuse or neglect, harassment, parole violations, and criminal trespass), are adequately described, with high *R*^2^, by regression models that do not include climate variables (*R*^2^ ≥ 0.5 in all cases), and only very small improvements can be gained by including climate effects when modeling robberies, burglaries, and theft. The positive association we find between temperature and aggressive crimes is in agreement with the associations found in previous studies [14, 15].

We find that aggravated assaults and batteries occurring on the street are more sensitive to climate variations than aggravated assaults and batteries occurring in residences, regardless of the time of year or the time of day. Days that are warmer than average significantly increase aggravated assaults and batteries in both locations, but the effect is more pronounced in street locations. The reduced effect of temperature on household aggravated assaults and batteries may be due to the fact that most residences these days are air conditioned [46], and this fact needs to be considered when comparing these results to the results of analyses conducted in the 1970’s or 1980’s.

Significant quadratic dependence of incidence of these crimes on temperature is only observed for aggravated assaults and batteries occurring in the street, and then only in spring and summer. From the coefficients in Table 6, we can estimate the temperature at which crime is most affected by temperature. If the relationship between crime incidence, *C*, and temperature, *T* is quadratic, we have

$$\begin{array}{c} C=a+bT+cT^{2}. \end{array}$$ (2)

The relationship is maximized when −*b* = 2*cT*, or *T* = −*b*/2*c*. From the coefficients for *b* and *c* for street aggravated assaults and batteries, shown in Table 6, we estimate that *T* = 89 ± 15 ∘F. This is in agreement with the results found, for example, in [14] and [34]. Other analyses that have not found a significant quadratic effect may have been limited by the number of crimes examined in their data, or the annual range of temperature at the locale considered [8, 10, 33].

Our finding that street assaults and batteries occur more often on warmer than average days is in concordance with previous research that has shown that aggression may be triggered by high temperatures [9–11, 13]. However, our additional finding of quadratic dependence of rates of assaults and batteries on temperature is also in concordance with those of other studies, which have hypothesized a Negative Effect Escape Model, which proposes that increasing discomfort from heat causes aggression to a point, but in very hot temperatures the motive to escape the heat becomes greater than the motive to aggress [47, 48].

We find no significant dependence of crime incidence on atmospheric pressure, in agreement with other studies [11]. We find that higher relative humidity only has a significant negative effect on aggravated assaults and batteries, and, in particular, only on aggravated assaults and batteries that occur in the street during the early evening hours. The effect is particularly pronounced on rainy days. This observation that high relative humidity appears to be negatively associated with crimes of aggression is in agreement with previous studies [19].

We find that wind also has a significant negative effect on aggravated assaults and batteries (and no significant effect on any other type of crime examined here). Again, the effect is only significant for aggravated assaults and batteries occurring in the street, not in residences, and the effect is most pronounced between noon and midnight.

Precipitation has a significant negative effect on aggravated assaults, batteries, theft and criminal damage. Just like relative humidity and wind, the negative effect is only significant for aggravated assaults and batteries occurring on the street, not in residences. The effect is most pronounced from noon to 5 PM, and then from 9 PM to 5 AM.

The observed negative influences of precipitation, high humidity, and high wind on assaults and batteries in the street may be further evidence of people being driven by inclement weather conditions from places where they may otherwise come into potential conflict with others, similar to the Negative Effect Escape Model, described above, that has been proposed to explain reductions in street crime at very high temperatures [47, 48].

Our analysis was confined to data from one location; the City of Chicago. Further analysis is needed of large crime data sets from other locales to determine if the magnitude of day-of-week, day-of-year, holiday, and climate effects on crime are unique to this locale, or differ in different climates or different societies. In particular, data from different latitudes would be useful in determination of whether or not the observed positive association of unusual fluctuations in assaults and batteries to unusual fluctuations in temperature is borne up in more tropical climates.

Not examined in our analysis was the potential of temporal changes in police vigilance perhaps contributing to the temporal trends observed in the data. Indeed, the likely influence of changing police enforcement standards on narcotics crime was the primary reason we did not examine this type of crime in this analysis (narcotics crime and prostitution are two types of crime likely most affected by changes in police vigilance and enforcement practices). However, our observation that there are quite different dependencies of different types of crime by day-of-week, day-of-year, holidays, and climate suggests that temporal changes in police enforcement are not the dominant cause of the temporal patterns in crime, which would tend to make the temporal changes more similar across types of crime.

In addition, not studied in this analysis is the potential confounding effects of geospatial heterogeneities in crime. For example, changes in local demographics, local intervention measures, etc can lead to spatial heterogeneities [49–51], and these spatial heterogeneities may mask important factors affecting local temporal dynamics [52]. While this can be studied by examining crime trends in finely granulated geospatial areas, fine granulation necessarily involves arbitrary choices of geospatial boundaries, which, as the granulation is made increasingly finer, increasingly restricts the size of the data sets within each of the areas. This in turn can mask important temporal trends due to the increased relative stochastic fluctuations in the small data sets, and presents serious problems from a predictive analytics perspective. Statistical methods are needed that combine temporal and geospatial information in a scale-invariant manner, while also taking account temporal trends and periodicity in the data (both weekday and annual). The recent work of [53] introduced a clustering method that takes into account geospatial information, and modeled potential annual periodicity in the data with a first order harmonic. Avenues of future research could potentially involve incorporating the more sophisticated temporal models used in this analysis with scale-invariant geospatial clustering methods, such as the novel Dynamic Covariance Kernel Density Estimation (DCKDE) method previously developed by the authors [2].

## Conclusions

In this analysis, we examined a sample of 5.7 million crimes in the City of Chicago over a 14 year period. We examined a wide array of exogenous factors that might be related to short term temporal trends in crime, including annual periodicity, holidays, day-of-week, paydays, and climate variables, including temperature, humidity, wind, air pressure, and precipitation. In our analysis, we examined various types of crime, corrected for auto-correlation in the data, and used bootstrapping methods for robust model selection. To our knowledge, ours is the first analysis of this type to correct for auto-correlation using harmonic regression methods, and to employ bootstrapping model selection methods that account for the multicollinearities in the explanatory variables. The analysis largely confirms the results of past studies examining the relationship of crime to these exogenous variables, particularly for a linear relationship between aggressive crime and temperature.

We take a predictive analytics approach in this analysis, and validate the predictive capability of our models. Model validation has been ignored in most (if not all) past studies, likely largely due to past studies testing the validity of various theories of crime, rather than having a focus on predictive analytics. From a predictive analytics perspective, a model that includes trend, annual variation, holidays, and weekdays has modest to good predictive capability for all types of crime considered in this analysis (model *R*^2^ ≥ 0.43 in all cases). This is in fact the model employed in the crime predictive analytics software described in [3] and [2].

The inclusion of temperature forecasts may be beneficial to the predictive capabilities of models in the short term for most types of crime. The additional inclusion of forecasts for precipitation and wind may be additionally beneficial to the short term predictive capabilities for assaults and batteries, but not other types of crime. However, the models in this study were based on retrospective data, and used the observed climate data on each particular day. Given that there are uncertainties inherent in climate forecasts, future studies would need to be done to assess the predictive reliability of such models when forecasts for climate variables are used; it may be that the uncertainties in climate forecast would degrade the improved model predictive power capabilities found in this study. In addition to climate, inclusion of the effects of holidays and school vacations may be beneficial, as well as inclusion of the longer term harmonic periodic patterns for different types of crime; such patterns may be location dependent within an urban area, and thus may be combined with geospatial analytics.
